# Neuromuscular blockade in acute respiratory distress syndrome: a systematic review and meta-analysis of randomized controlled trials

**DOI:** 10.1186/s40560-020-0431-z

**Published:** 2020-01-28

**Authors:** An Thi Nhat Ho, Setu Patolia, Christophe Guervilly

**Affiliations:** 1 Pulmonary and Critical Care Medicine, Saint Louis University, 3635 Vista Avenue, St Louis, MO 63104 USA; 2 Medical Intensive Care Unit, North Hospital, APHM, Marseille, France; 3 CEReSS, Center for Studies and Research on Health Services and Quality of Life EA3279, Aix-Marseille University, Marseille, France

**Keywords:** Neuromuscular blocking agent, Cisatracurium, Acute respiratory distress syndrome

## Abstract

**Background:**

Neuromuscular blocking agent (NMBA) has been proposed by medical guidelines for early severe acute respiratory distress syndrome (ARDS) because of its survival benefits. However, new studies have provided evidence contradicting these results.

**Method:**

A search was performed of the Pubmed, Scopus, Clinicaltrials.gov, and Virtual Health Library databases for randomized controlled trials (RCT) evaluating 28-day mortality in ARDS patients treated with NMBA within 48 h. An English language restriction was applied. Relevant data were extracted and pooled into risk ratios (RR), mean differences (MD), and corresponding 95% confidence intervals (CI) using random-effect model. Sensitivity and meta-regression analysis were performed.

**Results:**

From 2675 studies, we included five RCTs in the analysis, for a total of 1461 patients with a mean PaO_2_/FIO_2_ of 104 ± 35 mmHg. The cisatracurium group had the same risk of death at 28 days (RR, 0.90; 95% CI, 0.78–1.03; *I*^2^ = 50%, *p* = 0.12) and 90 days (RR, 0.81; 95% CI, 0.62–1.06; *I*^2^ = 56%, *p* = 0.06) as the control group (no cisatracurium). The secondary outcomes of mechanical ventilation duration and ventilator-free days were not different between the two groups. Cisatracurium had a significantly lower risk of barotrauma than the control group with no difference in intensive care unit (ICU)–induced weakness. The PaO_2_/FIO_2_ ratio was higher in the cisatracurium group but not until 48 h. Meta-regression analysis of the baseline PaO_2_/FIO_2_ ratio, positive end-expiratory pressure (PEEP) revealed no heterogeneity. Subgroup analysis excluding the trial using high PEEP and light sedation strategy yielded an improvement in all mortality outcomes.

**Conclusion:**

NMBA improves oxygenation only after 48 h in moderate, severe ARDS patients and has a lower barotrauma risk without affecting ICU weakness. However, NMBA does not reduce ventilator-free days, duration of mechanical ventilation or, most importantly, the mortality risk regardless of the severity of ARDS.

## Background

Acute respiratory distress syndrome (ARDS) is a severe diffuse lung injury characterized by significantly impaired oxygenation and lung compliance that frequently leads to mechanical ventilation [1–3]. ARDS is common, accounting for 10 to 15% of intensive care unit admissions and 200,000 patients a year in the US [4, 5]. In a large observational study, the mortality rate of moderate to severe ARDS was up to 40% [4]. Neuromuscular blocking agents (NMBA) have been proposed during the early course of moderate to severe ARDS especially in those with PaO_2_/FIO_2_ < 120 mmHg to improve oxygenation and reduce patient-ventilator dyssynchrony [6–8]. NMBA facilitates oxygenation in various studies [9–11] by various mechanisms, including reducing ventilator-induced lung injuries and inhibiting inflammatory cytokines [12, 13]. An early, short-term continuous infusion of cisatracurium, an NMBA, was evaluated in a large multicenter randomized study (ACURASYS trial), and evidence of its survival benefits in ARDS was found in an anticipated post hoc analysis of patients with PaO_2_/FIO_2_ < 120 mmHg. NMBA, however, could be associated with muscular weakness and the need of more sedatives [6, 14, 15]. Of note, besides NMBA use, increasing evidence of other supportive treatments for ARDS patients such as sedation, positive end-expiratory pressure (PEEP) strategy, and prone positioning has been developed since major data on NMBA were published [16–18]. The question of whether NMBA is effective is still unknown. Recently, the largest randomized controlled trial on NMBA in early ARDS patients was published to reassess the effect of an early, short-term continuous infusion of cisatracurium (ROSE trial), and no survival benefit was found [19]. This result was different from major studies published previously on the same topic [9–11].

Therefore, the objective of this study was to provide a comprehensive meta-analysis of data from all randomized controlled trials on neuromuscular blocking agent use in ARDS patients. We clarify the effects of NMBA on patient survival, oxygenation, and adverse events to potentially guide decisions among medical professionals treating ARDS patients.

## Methods

### Search strategy and study identification

We used the following electronic databases: Pubmed (1970 to present), Scopus (1970 to present), Clinicaltrials.gov (1970 to present), and Virtual health library (1970 to present) to identify relevant articles in July 2019. Search terms: (((((((ARDS[Title/Abstract]) OR acute respiratory distress syndrome[Title/Abstract]) OR respiratory distress syndrome, adult[MeSH Terms]) OR adult respiratory distress syndrome[MeSH Terms]) OR acute respiratory distress syndrome[MeSH Terms]) OR respiratory failure[Title/Abstract])) AND ((((((cisatracurium[Title/Abstract]) OR Neuromuscular blockade[Title/Abstract]) OR Neuromuscular blocking agent[Title/Abstract]) OR NMBA[Title/Abstract]) OR paraly*[Title/Abstract]) OR Nimbex[Title/Abstract]) were used. The study protocol followed the recommendations of the Preferred Reporting item for Systematic Review and Meta-analysis (PRISMA) statement [20]. A 2-level literature search was performed. A secondary search included manual scrutiny of the reference list of relevant articles received in the initial search after importing all searched studies from the databases into Endnote. English language restriction was applied.

### Study selection and data collection

We used the following inclusion criteria: randomized control trials (RCTs) in patients with adult respiratory distress syndrome (ARDS) who were randomized to receive neuromuscular blocker within the first 48 h of the diagnosis and who have evaluated 28-day mortality rate between the neuromuscular blocker group with the conventional treatment group. Studies were excluded if they were: (1) non-randomized controlled trials, (2) conference posters, and (3) did not measure mortality outcomes. Any difference in opinion about the eligibility was solved by discussion and consensus.

### Full-text screening and data extraction

Two reviewers extracted the data independently into a spreadsheet. The data sought included (1) study characteristics (title, authors, year of publication, study location, number of patients, age, important exclusion criteria); (2) baseline patients demographic and clinical characteristics (age, sex, baseline fractional inspiratory oxygen (FIO_2_), PEEP, PaO_2_/FIO_2_ ratio, tidal volume, causes of ARDS, sepsis scores); (3) mortality at 28 days, 90 days, ICU mortality, mechanical ventilation duration, ventilator-free days at day 28, days not in ICU at day 28; (4) adverse events (Medical Research Council scale, ICU weakness at day 28, barotrauma). Barotrauma was defined as either newly developed pneumothorax, pneumomediastinum, or subcutaneous emphysema. Any disagreement between the two reviewers was resolved by consensus.

### Endpoints

The primary endpoint of this meta-analysis was the 28-day mortality relative risk between the NMBA interventional group and the control group. The secondary endpoint was the 90-day ICU mortality relative risks, the difference in mechanical ventilation duration, ventilator-free days at day 28, days not in ICU at day 28, and the adverse events.

### Risk of bias and evidence profile

We used the Cochrane Risk of Bias tool for a randomized control trial to assess the bias profile of individual trials [21]. The tool includes 7 domains: random sequence generation, allocation concealment, blinding of participants and personnel, blinding of outcome assessment, incomplete outcome data, selective reporting, and other possible bias. Risks were classified as low, high risk, or unclear. Publication bias was analyzed using Egger’s regression test and funnel plot. The quality of the evidence was assessed for the primary endpoint in agreement with the Grading of Recommendations Assessment Development and Evaluation system [22].

### Statistical analysis

Statistical analyses were performed using the Review Manager 5.3 software (Cochrane Collaborative Oxford, UK) and Comprehensive Meta-Analysis 2.0 software. Individual study relative risks (RRs) and 95% CI were calculated for dichotomous outcomes and mean differences (MDs) with 95% CI were estimated for continuous outcomes. Summary estimates of RRs were determined using the DerSimonian and Laird random-effects models. The variability across studies not by chance was tested using the *I*^2^ statistics and *p* > 0.05 was considered not statistically significant. Inconsistency across the studies was classified as low if 25% < *I*^2^ ≤ 50%, moderate if 50% < *I*^2^ ≤ 75%, and high if *I*^2^ > 75 [23]. Additional sensitivity analyses were performed to evaluate the effect of key assumptions on the overall results or the robustness of the overall results. We further form meta-regression models to assess for potential heterogeneity for the following factors: PaO_2_/FIO_2_ ratio (P/F ratio), baseline PEEP, predicted mortality, PEEP strategy, and prone positioning. Predicted mortality was based on available studies on SOFA score (first in full text) and SAPS II (first in full text) score [24–26]. Publication bias was analyzed as previously described by the funnel plot and regression test for funnel plot asymmetry. A *p* value of < 0.05 was considered a statistically significant publication bias.

## Results

### Literature search results and study characteristics

**Fig. 1:**
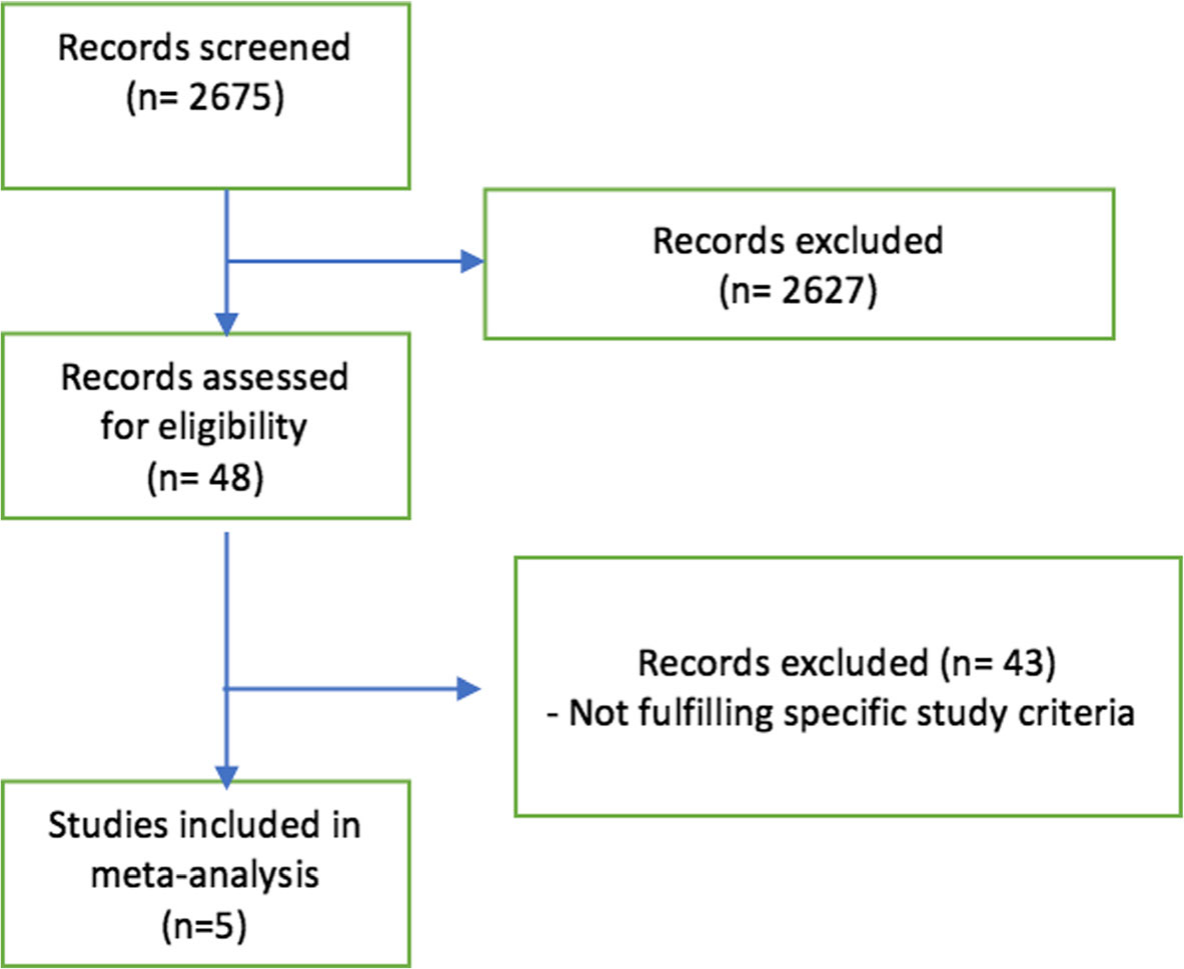
Screening and selection process flow diagram

Using the search terms mentioned above, we identified 2675 studies. After screening the study abstracts of the above studies, five studies met the inclusion criteria for comparing mortality in patients with ARDS treated with an additional NMBA vs conventional treatment groups. Five studies were included for a full-text review [9–11, 19, 27]. The flow diagram of the study selection process is shown in Fig. 1.

A description of the studies included in our analysis is shown in Table 1. A total of 1462 patients were included in the five trials. All of the studies included patients within 48 h of the ARDS diagnosis with a cut-off PaO_2_/FIO_2_ ratio mostly below 150 mmHg with the exception of Forel et al. (cut-off below 200). Guervilly et al. only randomized patients with moderate ARDS and PaO_2_/FIO_2_ between 100 and 150 mmHg. The pooled data for the baseline respiratory parameters had a PEEP (cmH_2_0) of 11.68 ± 3.74 and a P/F ratio of 104 ± 32.50. The baseline demographic and respiratory parameters (PEEP, PaO_2_/FIO_2_ ratio) were not statistically significantly different between the NMBA group and the control group. The ARDS causes mostly include pneumonia (59%), followed by aspiration, extrapulmonary sepsis, other causes, and lung contusion (17%, 14%, 9%, and 1% respectively).

**Table 1:** Characteristics of included trials

Trial author or trial name	Gainnier et al. [10]	Forel et al. [9]	ACURASYS trial [11]	Guervilly et al. [27]	ROSE trial [19]
Year	2004	2006	2010	2017	2019
Country	France	France	France	France	USA
NBMA vs control (patients)	28 vs 28	18 vs 18	177 vs 162	13 vs 11	501 vs 505
PaO_2_/FIO_2_ ratio and PEEP for inclusion (cm H_2_O)	< 150 > 5	< 200	< 150 > 5	100–150 > 5	< 150 > 8
Important exclusion criteria	NMBA use within 2 weeks	NMBA and steroids use within 2 weeks	Current continuous infusion of NMBA	Current continuous infusion of NMBA	Current continuous infusion of NMBA
Ventilator management strategy	Lung protective ARDS net protocol	Lung protective ARDS net protocol	Lung protective ARDS net protocol	Lung protective ARDS net protocol	Lung protective high PEEP strategy
Cisatracurium dose	Bolus of 50 mg, 5 μg/kg/min	Bolus 0.2 mg/kg, 5 μg/kg/min	Bolus of 15 mg, 37.5 mg/hour	Bolus of 15 mg, 37.5 mg/hour	Bolus 15 mg, 37.5 mg/hour
Assessment for an extra dose	Train-of-four	Train-of-four	Plateau pressure > 32	Train-of-four	No adjustment
Sedation goal	Ramsay 6	Ramsay 6^b^	Ramsay 6	Ramsay 6	RASS − 4, − 5 in the intervention group RASS 0 to − 1 in the control group)^c^
Risk of bias					
Sequence generation	Low	low	Low	Low	Low
Allocation concealment	Low	Low	Low	Low	Low
Blinding	High^a^	High^a^	Low	High^a^	High^a^
Withdrawal	Low	Low	Low	Low	Low
Selective outcome	Low	Low	Low	Low	Low
Free of other bias	Low	Low	Low	Low	Low
Overall	High	High	Low	High	High

^a^Risk was high because nurses are not blinded to the intervention group

^b^Ramsay scale (1–6) 6 no response [28]

^c^RASS score (+ 4 to − 4) 0 to − 1: alert and calm, drowsy [29]

All of the studies followed the ARDS net protocol lung protective strategy for ventilator management. Cisatracurium bolus and infusion was used as the NBMA agent with the bolus and continuous infusion doses varying across studies. The most recent two large RCT by Moss and Papazian did not use train-of-four nerve stimulation for additional boluses and cisatracurium was given as a fixed-dose continuous infusion. All of the studies were considered high risk for bias because of the lack of blinding to the clinical nurses or other healthcare professionals, except for the trial by Papazian et al.

### Mortality outcomes

**Fig. 2:**
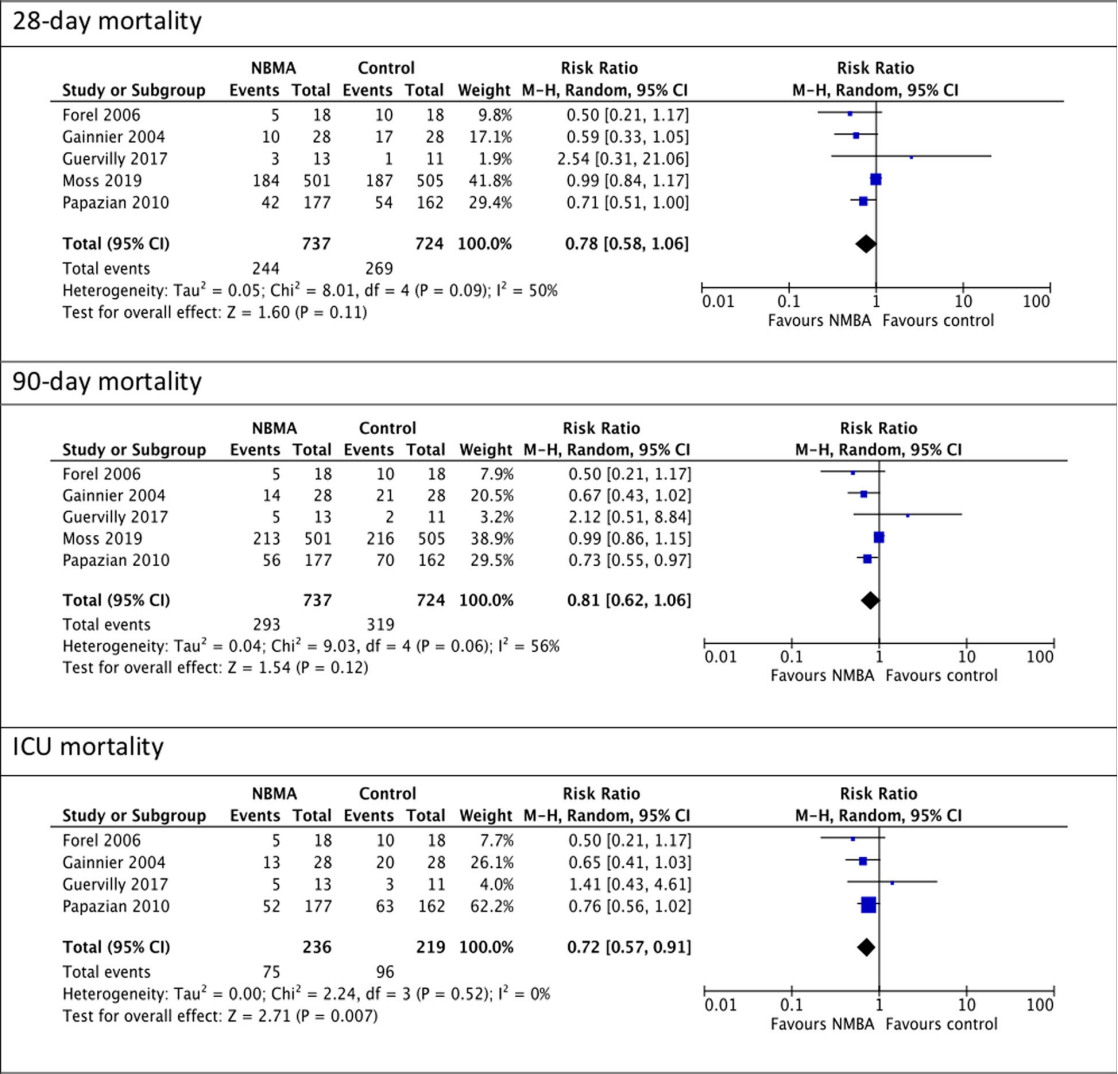
Mortality outcomes

The mortality outcomes are presented in Fig. 2. The cisatracurium group had the same risk of death at 28 days (RR, 0.90; 95% CI, 0.78–1.03; *I*^2^ = 50%, *p* = 0.12) and 90 days (RR, 0.81; 95% CI, 0.62–1.06; *I*^2=^ 56%, *p* = 0.06) but had a significantly lower ICU mortality compared to the control group (RR, 0.72; 95% CI, 0.57–0.91; *I*^2^ = 0%; *p* = 0.007). It was noted that the ICU mortality pooled RR did not include the ROSE trial.

### Other outcomes

**Fig. 3:**
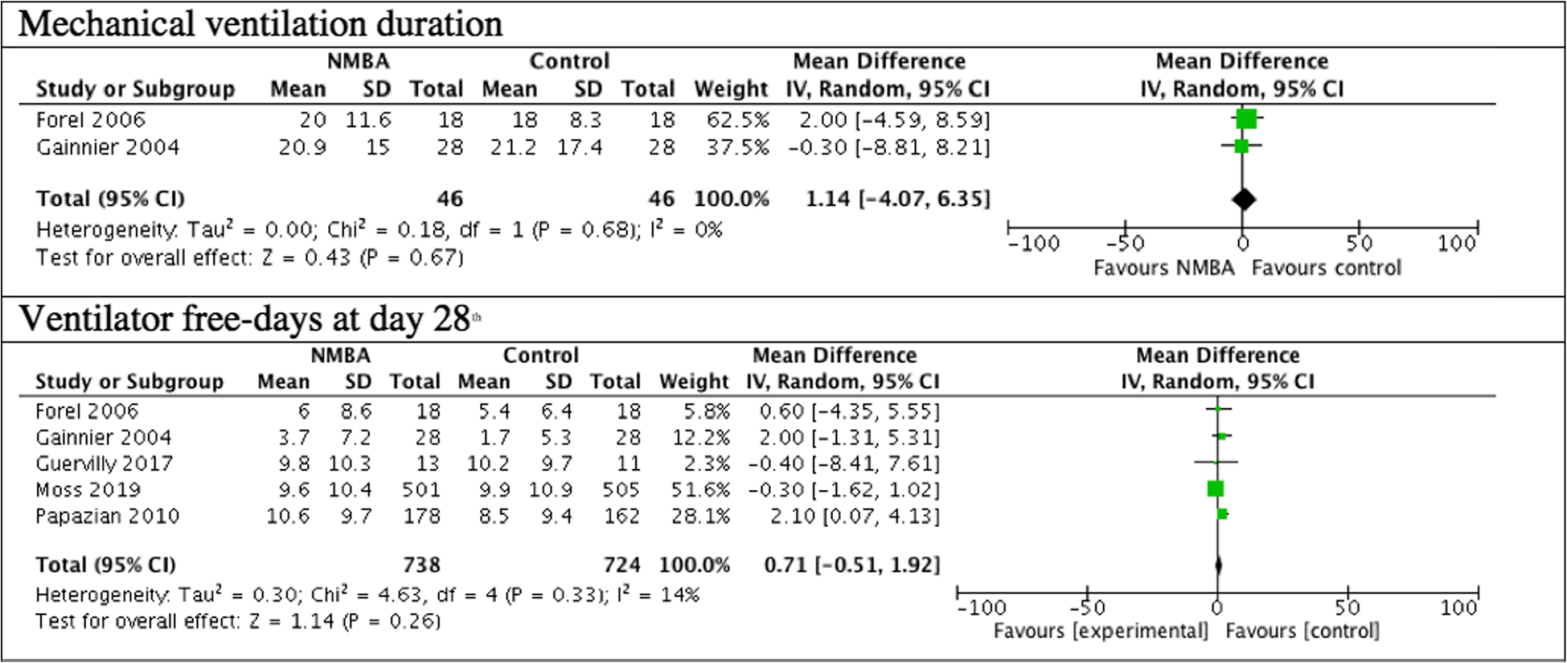
Mechanical ventilation duration and ventilator-free days truncated at day 28th outcomes

The mechanical ventilation duration was measured in two studies totaling 92 patients and ventilator-free days at day 28 were measured by five trials totalizing 1462 patients. The secondary outcomes of mechanical ventilation duration and ventilator-free days were not different between the cisatracurium group and the control group (Fig. 3).

### Adverse events

**Fig. 4:**
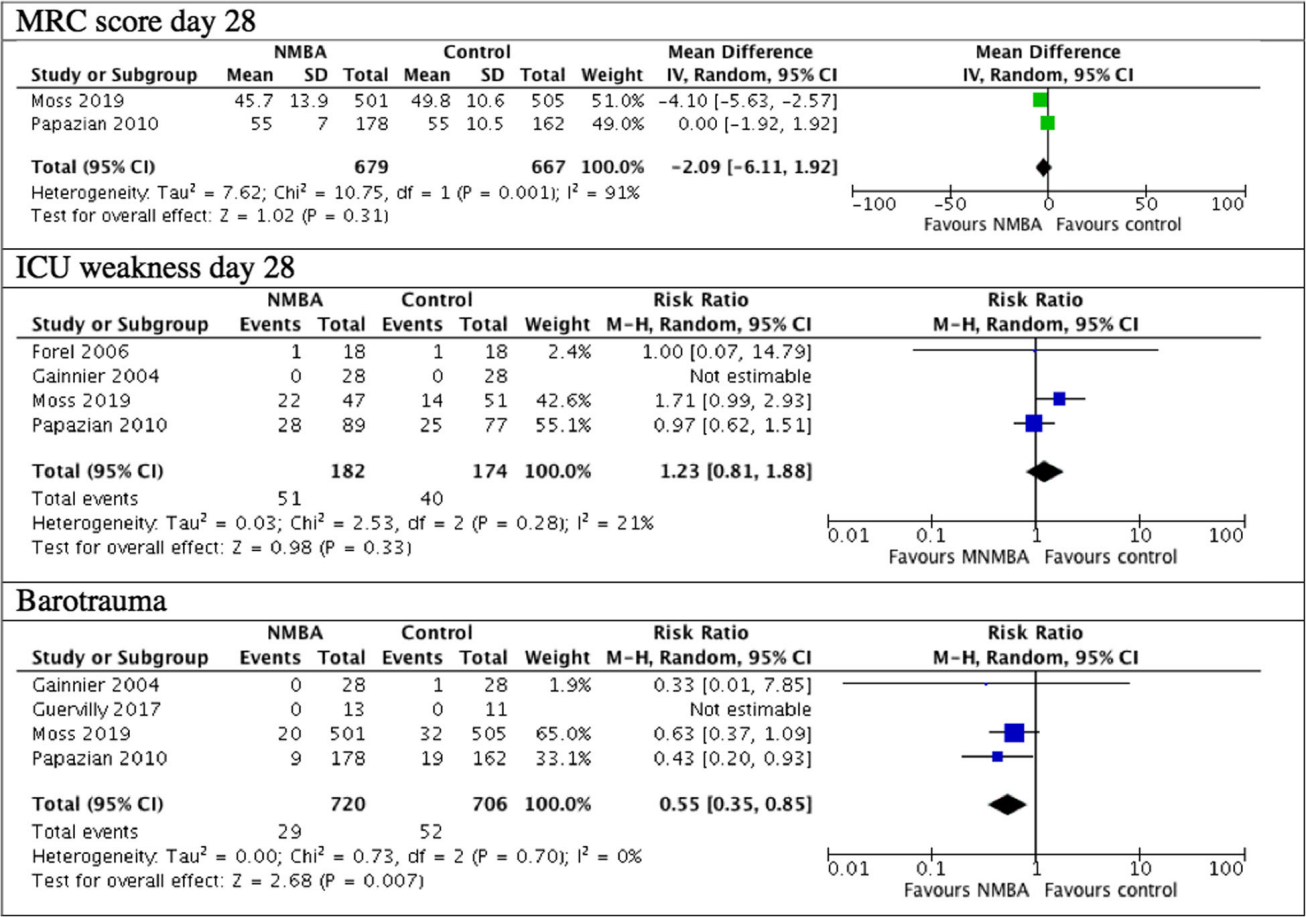
Adverse events

The cisatracurium group had a statistically significant lower risk of barotrauma than the control group (RR, 0.55; 95% CI 0.35–0.85, *I*^2^ = 0%, *p* = 0.007). Four trials reported ICU weakness. There was no difference in the risk of ICU weakness at day 28 between the two groups (RR, 1.23; 95% CI, 0.81–1.88; *I*^2^ = 21%; *p* = 0.33). The Medical Research Council (MRC) scale was used in 2 studies to evaluate ICU weakness and was not significantly different between the two groups (see Fig. 4).

### Oxygenation and plateau pressure

**Fig. 5:**
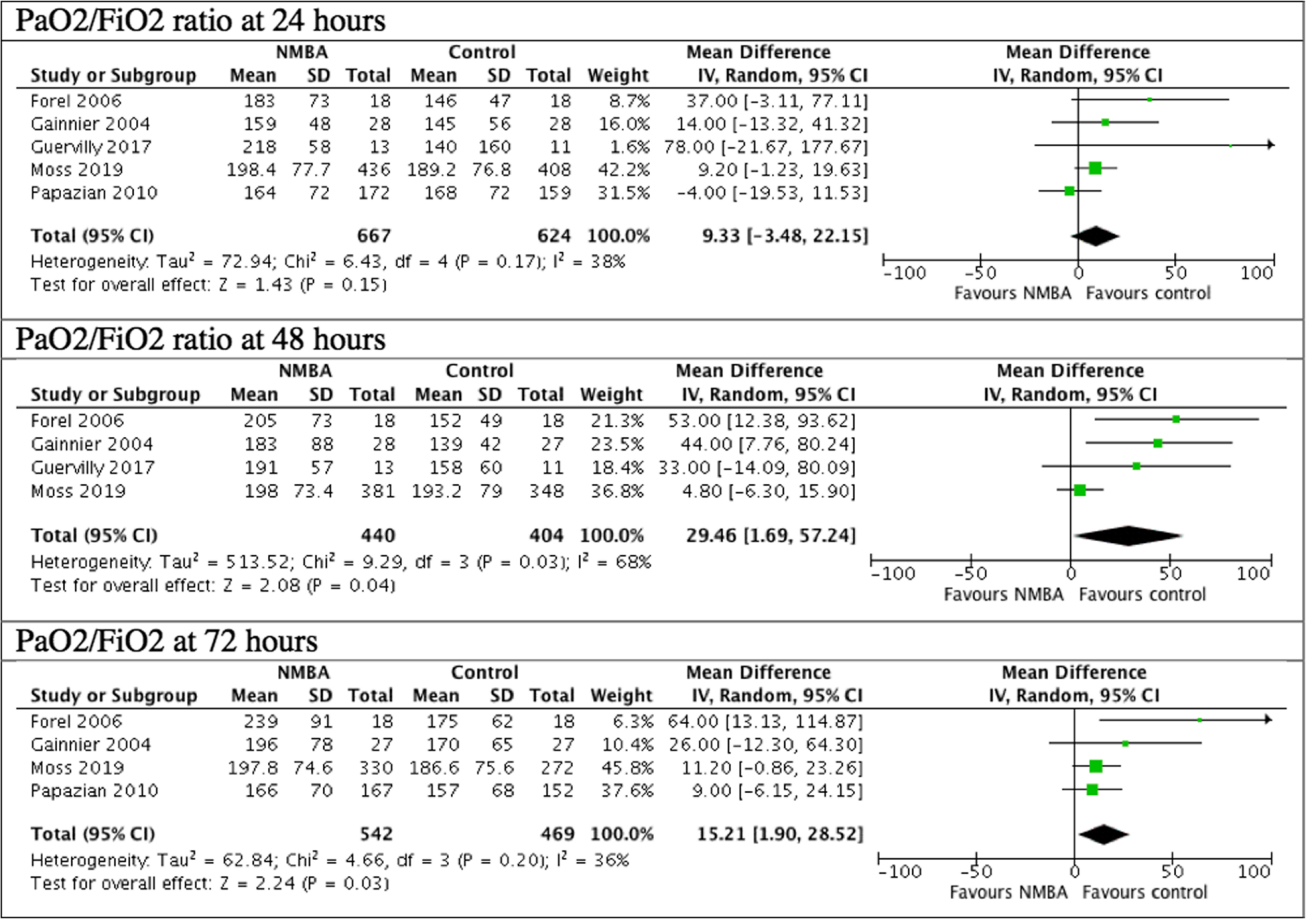
P/F ratio

The comparison of the PaO_2_/FIO_2_ ratio between the NMBA group and the control group showed a statistically significant difference at 48 h and at 72 h ((MD, 29.46; 95% CI 1.69–57.24, *I*^2^ = 68%, *p* = 0.04; MD, 15.21; 95% CI, 1.9–28.52, *I*^2^ = 36%, *p* = 0.03). From 0 to 24 h, there was no difference noted between the NMBA group and the control group (*p* > 0.05) (Fig. 5). There were only two studies, totaling 596 patients, reporting PaO_2_/FIO_2_ ratio on day 7 [11, 19]. PaO_2_/FIO_2_ ratio was higher in the intervention group (MD, 12.97; 95% CI, 0.26, 25.68, *I*^2^ = 0%, *p* = 0.05).

**Fig. 6:**
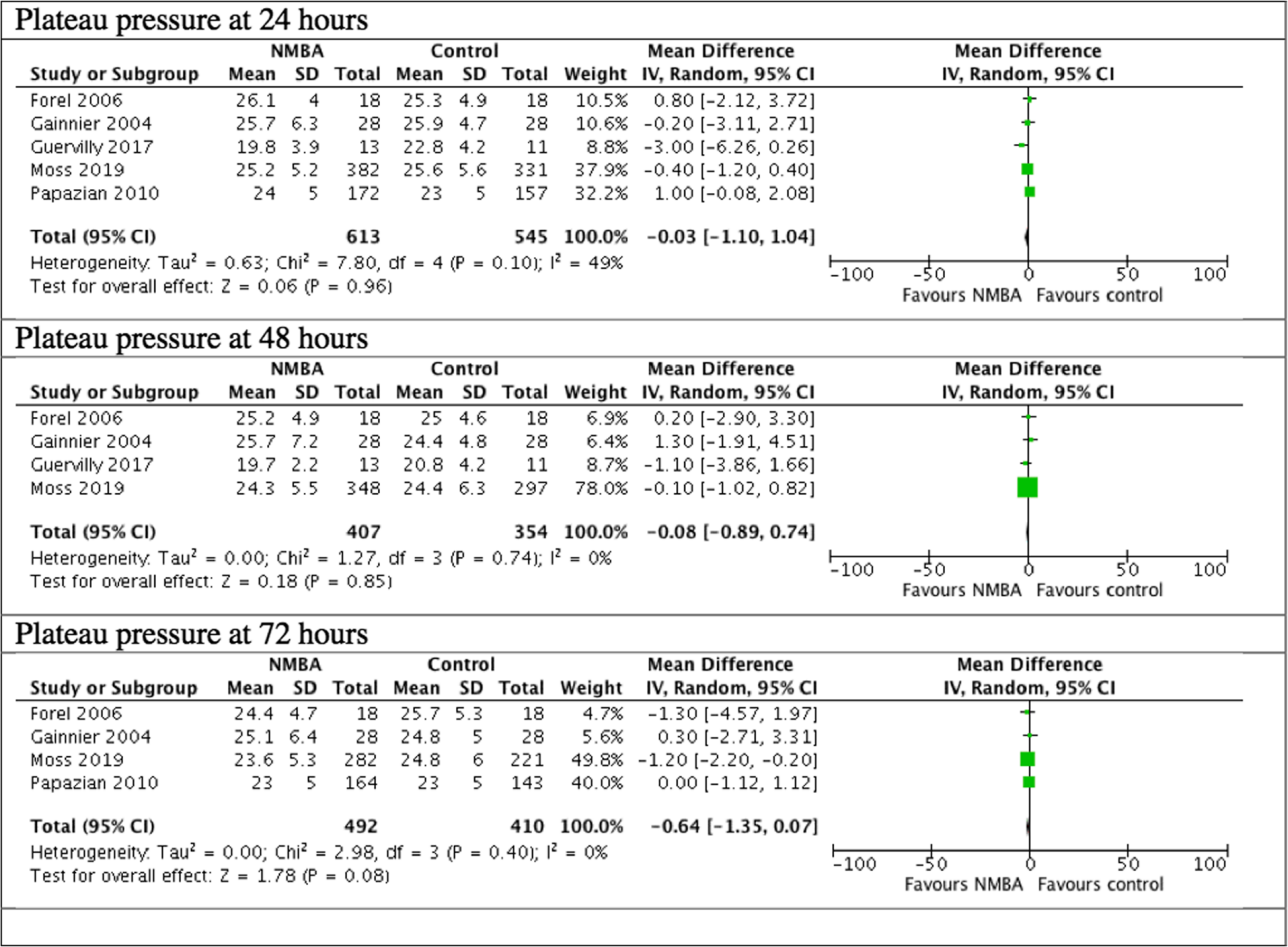
Plateau pressure

There was no difference in the plateau pressure between the NMBA group and the control group in 24 h, 48 h, or 72 h (Fig. 6).

### Sensitivity analysis, subgroup analysis, and meta-regression

A sensitivity analysis was performed for all outcomes by changing the statistical measure to odds ratios or risk differences did not show a change in results. A meta-regression analysis investigating the potential effects of baseline PaO_2_/FIO_2_, PEEP, prone positioning rate, and overall mortality of the control group revealed no significant relationship between those factors and 28-day mortality (*p* values = 0.42, 0.58, 0.55, 0.19).

**Fig. 7:**
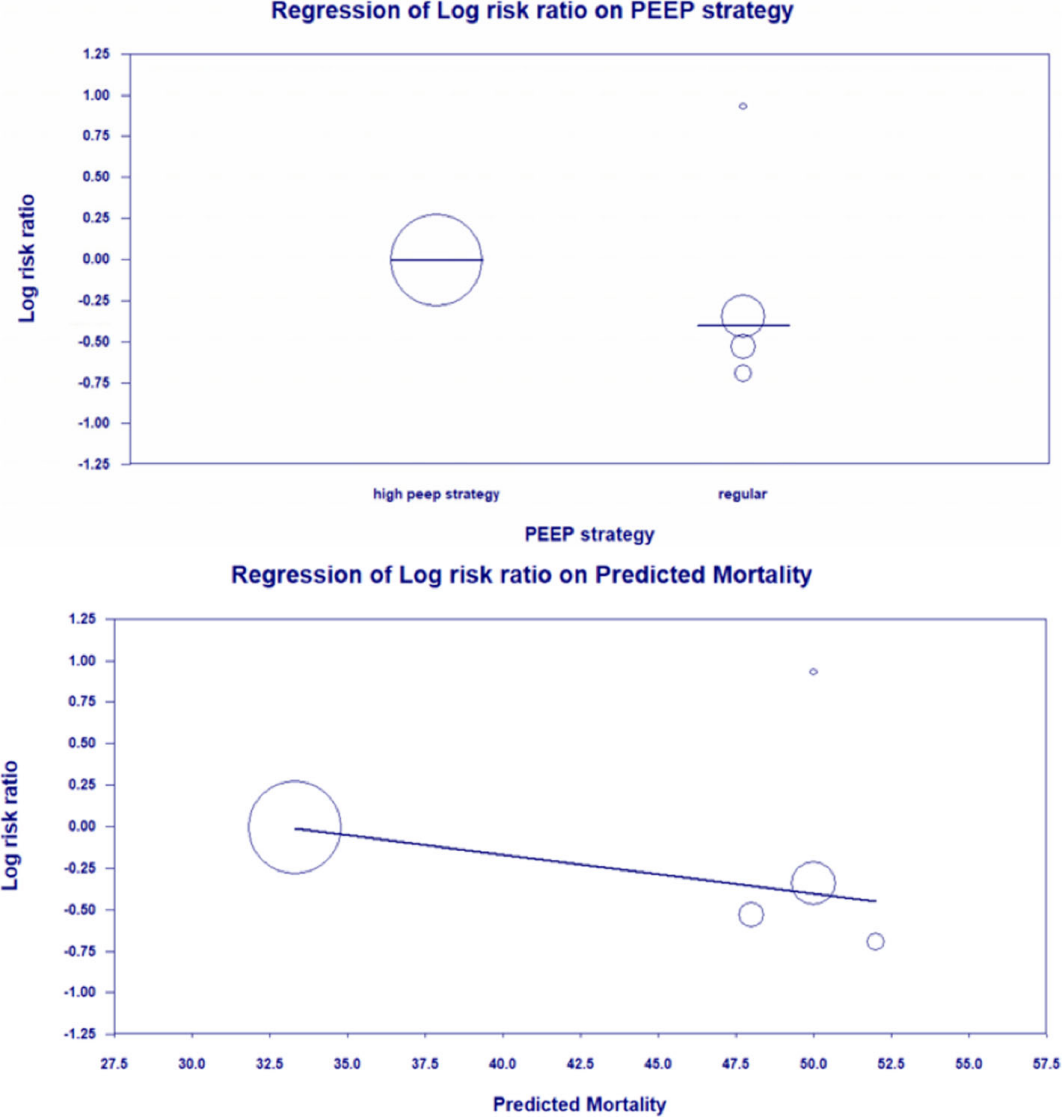
Regression of Log risk ratio on PEEP strategy and predicted mortality

Similarly, a meta-regression analysis was performed to evaluate the changes in predicted mortality on day 1 based on the disease severity grading system (SAPS II and SOFA score). This analysis found a significant effect of predicted mortality on the relationship between 28-day mortality and NMBA use (*p* = 0.0197, see Fig. 7).

A subgroup analysis of PEEP strategy that excluded the study using the high PEEP strategy by Moss et al. yielded a difference in all mortality outcomes with the NMBA group having significantly lower mortality at 28 days, at 90 days, and in the ICU. The statistical measure for 28-day mortality was RR 0.66, 95% CI 0.50–0.87, *I*^2^ = 58%, and *p* = 0.003; for 90-day mortality was RR 0.69, 95% CI 0.55–0.87, *I*^2^ = 0%, and *p* = 0.001; and for ICU mortality was RR 0.72, 95% CI 0.57–0.91, *I*^2^ = 0%, and *p* = 0.006.

### Publication bias

We carried out Egger’s regression test and funnel plots to assess the presence of publication bias. The funnel plots showed no clear evidence of asymmetry and Egger’s regression test indicated no evidence of publication bias (data not shown).

### Quality of evidence

The quality of evidence is found in Table 2.

**Table 2:** Quality of evidence assessment

Question	Quality assessment				Quality of evidence
	Bias	Inconsistency	Indirectness	Imprecision	
28-day mortality	Serious^a^	Moderate^b^	No serious indirectness	No serious imprecision	Moderate
90-day mortality	Serious^a^	Moderate^b^	No serious indirectness	Undetected	Moderate
ICU mortality	Serious^a^	Undetected	Undetected	Detected^c^	Low
Mechanical ventilation duration	Serious^a^	Undetected	No serious indirectness	No serious imprecision	Moderate
Ventilator-free days at day 28th	Serious^a^	Undetected	No serious indirectness	No serious imprecision	Moderate
ICU weakness	Serious^a^	Undetected	No serious indirectness	No serious imprecision	Moderate
Barotrauma	Serious^a^	Undetected	No serious indirectness	No serious imprecision	Moderate

^a^Bias was rated high because of no blinding in four out of five studies (see Table 1)

^b^Inconsistency across studies in these two outcomes based on *I*^2^ was moderate

^c^Imprecision was detected for this outcome because there was no value for this outcome available for 1 out of 5 studies

## Discussion

In our study, we performed a comprehensive analysis of the most current available data on the effects of neuromuscular blockers on outcomes in patients with ARDS. We reviewed all good-quality randomized controlled trials performed over the past 15 years. The main finding of our study was that neuromuscular blocker, specifically cisatracurium did not lead to a reduction in the mortality risk at 28 days and 90 days for ARDS patients. Additionally, there was no reduction of ventilator-free days or duration of mechanical ventilation. NMBA improved oxygenation but not until 48 h and was associated with lower barotrauma but did not increase the risk of ICU weakness. The mortality risk at 28 days and 90 days was not affected by prone positioning rate, baseline P/F ratio, or PEEP but was affected by the PEEP strategy and predicted mortality risk before randomization.

Neuromuscular blockers have long been used for severe ARDS to control patient-ventilator dyssynchrony and allow a tolerance of permissive hypercapnia [30]. Since the three randomized controlled trials on the effects of neuromuscular blocker totaling 431 showed survival benefits dating back before 2010, the clinical guidelines have recommended NMBA as supportive treatment of severe ARDS. The recommendation strength was only weak to moderate due to the lack of blinding and the risk of indirectness in the contributing studies [6, 8, 31]. There are also still concerns about ICU-related weakness and other risks associated with NMBA, such as atelectasis, diaphragm paralysis, and anaphylaxis.

A meta-analysis of the mortality outcomes at 28 and 90 days did not show a statistically significant different risk between the group given NMBA and control group. Subgroup analysis of all studies excluding the ROSE trial by Moss et al. showed a reduction in all mortality outcomes in the NMBA group. ROSE was the first trial on the topic performed not in France but in the US. The authors used a few different methods compared to other trials. For example, the control arm of Moss et al. included 84 (17%) patients receiving NMBA which was different than other studies and may have reduced the mortality of the control arm more than expected. A meta-regression analysis also showed that the heterogeneity in the outcome measure was due to the predicted mortality, sedation strategy and PEEP strategy which were the major differences between ROSE and other studies.

Furthermore, Moss et al. used a modified PEEP table from the ALVEOLI trial while the other four trials used the protocol proposed by the National Institutes of Health for the ARDS group [32]. The high PEEP strategy in the ROSE study applied higher PEEP than the regular strategy used in other trials (see Additional file 1 for the PEEP strategies). It was noted that regardless of the implementation of high peep, the barotrauma incidence in the ROSE trial was the same as the average barotrauma incidence in the remaining studies (5.1% vs 6.9%, *p* = 0.2). In addition, Moss et al. used a light sedation strategy (RASS score of 0 to − 1, alert and calm to drowsy) for the control group while other trials aimed for a RAMSAY score of 6 (no response) for both the intervention and control group. This light sedation strategy in the control group could explain the primary outcome difference between the ROSE study and other trials. Light sedation was associated with less use of vasopressor, shorter time to extubation [33], and lower mortality in mechanically ventilated patients [17, 18].

The meta-regression showed that the baseline PaO_2_/FIO_2_ ratio and baseline PEEP variables had no effect on the 28-day mortality rate and NMBA. This finding is a new concept because the ACURASYS trial showed a mortality benefit for NMBA use in ARDS patients with a P/F ratio less than 120 [11]. Prone positioning was shown to improve mortality in severe ARDS in available literature [16, 34, 35]. In this analysis, we do not have sufficient data to draw the conclusion on whether or not the cisatracurium besylate infusion was more effective when combined with prone positioning. However, the percentage of patients who were proned does not affect the relationship between mortality and NMBA.

Our study showed a positive effect of NMBA on oxygenation with a decreased PaO_2_/FIO_2_ ratio observed after 48 h, which was consistent with earlier evidence [36]. NMBA’s positive effect on oxygenation could be related to the decrease in ventilator-induced lung injury associated with barotrauma. The risk of barotrauma was lower in the NMBA group compared to the control group when we combined the effects of the four studies reporting this outcome. Moreover, NMBA also had an anti-inflammatory effect and decreased oxygen consumption by active muscle groups [9, 30]. However, NMBA had this positive effect on oxygenation only after 48 h and did not improve plateau pressure at 24 to 72 h.

Cisatracurium was not associated with the risk of ICU-acquired weakness. Although neuromuscular blocker has been widely used, there is still a concern regarding their use in clinical practice [37], especially when concomitant use of corticosteroids for severe septic shock could have an additive effect with NMBA [14]. Our finding of no risk of ICU-acquired weakness is likely because of the short duration of neuromuscular blocker of only 48 h in all the studies. Of note, only one study by Gainnier et al. did not use corticosteroids in the first 5 days. Other studies used corticosteroids in 5 to 45% for septic shock but there is no difference between the corticosteroids use when we compare between the NMBA group and the control group (OR 1.13; 95% CI, 0.89–1.44; *p* = 0.14).

The limitation of the quality of the evidence in this analysis included the bias from the unblind healthcare professionals in almost all studies. The largest trial also gave cisatracurium to a percentage of patients in the control group and was stopped early due to futility before a sample size with an adequate power was reached. Regardless, trials that stopped early for benefit do not have strong evidence of causing a substantial bias in the evaluation of the treatment effects [38]. Because of these limitations, the quality of evidence was judged to be only moderate. The second limitation was that we could not obtain the ICU mortality in the study of Moss et al. so we could not draw a conclusion about this particular outcome.

Despite these limitations, our study comprehensively reviewed all available randomized controlled trials for evidence of the use of neuromuscular blockers in ARDS patients. The clinical management of ARDS patients is still a complicated field that considers multiple other factors. In clinical practice, NMBA still has a benefit of improving oxygenation and reducing barotrauma without increasing ICU weakness. However, it is noted that this benefit of oxygenation is not observed until 48 h. In fact, NMBA may not be necessary for the purpose of mortality reduction, when there is an implementation of high PEEP with appropriate protocols to decrease PEEP, light sedation in combination with prone positioning and low tidal volume ventilation.

## Conclusion

NMBA did not lead to a reduction in the mortality risk of 28 days and 90 days, ventilator-free day, duration of mechanical ventilation for moderate/severe ARDS patients. NMBA improved oxygenation but not until 48 h and was associated with lower barotrauma without affecting ICU weakness. The mortality risk of 28-day with NMBA was not affected by the baseline severity of ARDS.

## Supplementary information

**Additional file 1.** PEEP table.

## Data Availability

The datasets used for the current study are available from the corresponding author on reasonable request.

## Abbreviations

ARDS: Acute respiratory distress syndrome
NMBA: Neuromuscular blocking agents
PEEP: Positive end-expiratory pressure
RCT: Randomized controlled trial
RR: Relative risk
