# Supplementary material for: Neuromuscular blockade in acute respiratory distress syndrome: a systematic review and meta-analysis of randomized controlled trials
Source: J Intensive Care. 2020 Jan 28;8:12. doi: 10.1186/s40560-020-0431-z (PMC6986163; doi:10.1186/s40560-020-0431-z)
Supplement: Supplementary file 1 — Additional file 1. PEEP table. [file 40560_2020_431_MOESM1_ESM.docx]

**PEEP table**

| FiO_2_ | PEEP in ROSE study  (cm of water) | PEEP in other studies  (cm of water) |
| --- | --- | --- |
| 0.3 | 5 | 5 |
| 0.4 | 5-16 | 5 |
| 0.5 | 16-20 | 8-10 |
| 0.6 | 20 | 10 |
| 0.7 | 20 | 10-14 |
| 0.8 | 20-22 | 14 |
| 0.9 | 22 | 14-18 |
| 1 | 22-24 | 18-24 |
